# Supplementary material for: Survey of Serum Amyloid A and Bacterial and Viral Frequency Using qPCR Levels in Recently Captured Feral Donkeys from Death Valley National Park (California)
Source: Animals (Basel). 2020 Jun 23;10(6):1086. doi: 10.3390/ani10061086 (PMC7341296; doi:10.3390/ani10061086)
Supplement: Supplementary file 1 [file animals-10-01086-s001.zip › Table S1.docx]

**Table S1.** Summary of the results for the partially overlapping samples t-test with Welch’s degrees of freedom to determine differences in the means in categories within clinical examination signs, SAA maximal concentration expressed in mg/L, microbial loads qPCR-assay and controls between sampling moment one and two.

|  | **Parameters** | **t-statistic** | **p-value** | **Estimate difference in the means** | **Confidence interval** |
| --- | --- | --- | --- | --- | --- |
| Clinical Examination Signs | Body Condition Score | 5.11 | 0.01 | 0.69 | 0.69 ; 0.70 |
|  | Behaviour signs | 2.28 | 0.03 | 0.15 | 0.14 ; 0.15 |
|  | Skin/Hair condition | 1.53 | 0.13 | 0.29 | 0.28 ; 0.31 |
|  | Lameness presence | -0.93 | 0.36 | -0.04 | -0.04 ; -0.03 |
|  | Ocular discharge presence | -0.80 | 0.43 | -0.05 | -0.06 ; -0.05 |
|  | Nasal discharge presence | 2.90 | 0.01 | 0.24 | 0.24 ; 0.25 |
|  | Abnormal breathing presence | -1.14 | 0.26 | -0.05 | -0.06 ; -0.05 |
|  | Coughing presence | 1.05 | 0.01 | 0.80 | 0.79 ; 0.81 |
| SAA | SAA maximal concentration (mg/L) | -0.98 | 0.33 | -6.62 | -7.04 ; -6.19 |
| Pathogen load qPCR-assay | Asinine Herpesvirus 2 aka EHV7 | 3.00 | 0.01 | 1.66 | 1.62 ; 1.69 |
|  | Asinine Herpesvirus 3 aka EHV8 | -0.72 | 0.47 | -0.20 | -0.21 ; 0.18 |
|  | Asinine Herpesvirus 5 | 1.81 | 0.08 | 1.74 | 1.68 ; 1.80 |
|  | Equine Herpesvirus 1 | N/A | 1.00 | 0.00 | N/A |
|  | Equine Herpesvirus 1 neuropathogenic | N/A | 1.00 | 0.00 | N/A |
|  | Equine Herpesvirus 1 nonneuropathogenic | N/A | 1.00 | 0.00 | N/A |
|  | Equine herpesvirus 4 | N/A | 1.00 | 0.00 | N/A |
|  | *Streptococcus equi* subspecies *equi* | -0.74 | 0.46 | -0.06 | -0.06 ; -0.05 |
|  | *Streptococcus equi* subspecies *zooepidemicus* | 2.42 | 0.02 | 2.00 | 1.95 ; 2.05 |
|  | Influenza AH3N8 | N/A | 1.00 | 0.00 | N/A |
|  | Equine rhinitis A virus | N/A | 1.00 | 0.00 | N/A |
|  | Equine rhinitis B virus | N/A | 1.00 | 0.00 | N/A |
| Controls | Glyceraldehyde 3 phosphatedehydrogenase (First control) | 6.48 | 0.01 | 5.63 | 5.57 ; 5.68 |
|  | Glyceraldehyde 3 phosphatedehydrogenase (Second control) | 2.97 | 0.01 | 2.77 | 2.71 ; 2.82 |
| N/A: Not applicable as all animals reported the same values; Welch's degrees of freedom: 70.01. | | | | | |
