# Supplementary material for: Survey of Serum Amyloid A and Bacterial and Viral Frequency Using qPCR Levels in Recently Captured Feral Donkeys from Death Valley National Park (California)
Source: Animals (Basel). 2020 Jun 23;10(6):1086. doi: 10.3390/ani10061086 (PMC7341296; doi:10.3390/ani10061086)
Supplement: Supplementary file 1 [file animals-10-01086-s001.zip › Table S2.docx]

**Table S2.** Model summary of stepwise linear regression for Asinine Herpesvirus (AHV-2) and *Streptococcus* *equi* subspecies *zooepidemicus* qPCR-assay.

| **Variable** | **R** | **R Square** | **Adjusted R Square** | **P-value** |
| --- | --- | --- | --- | --- |
| Asinine Herpesvirus (AHV-2) | 0.453 | 0.205 | 0.144 | 0.005 |
| *Streptoccocus equi* subspecies *zooepidemicus* | 0.390 | 0.152 | 0.087 | 0.040 |
