# Supplementary material for: Survey of Serum Amyloid A and Bacterial and Viral Frequency Using qPCR Levels in Recently Captured Feral Donkeys from Death Valley National Park (California)
Source: Animals (Basel). 2020 Jun 23;10(6):1086. doi: 10.3390/ani10061086 (PMC7341296; doi:10.3390/ani10061086)
Supplement: Supplementary file 1 [file animals-10-01086-s001.zip › Table S3.docx]

**Table S3.** Standardized Coefficients for factors presenting a significant relationship with Asinine Herpesvirus (AHV-2) and Streptococcus *equi* subspecies *zooepidemicus* q-PCR microbial loads and significance within each predictive CATREG model.

|  | **Variable** | **Beta (β) Standardized Coefficients** | **Bootstrap (1000)**  **Estimate of Std. Error** | **Degrees of freedom (df)** | **F** | **P-value** |
| --- | --- | --- | --- | --- | --- | --- |
| Asinine Herpesvirus (AHV-2) | BCS (1-5) | -0.055 | 0.178 | 1 | 0.097 | 0.757 |
|  | Behaviour signs | 0.406 | 0.269 | 2 | 2.281 | 0.109 |
|  | Nasal discharge presence | 0.171 | 0.09 | 1 | 3.571 | 0.063 |
|  | Coughing presence | 0.035 | 0.054 | 1 | 0.413 | 0.522 |
|  | Sampling moment | 0.012 | 0.116 | 1 | 0.011 | 0.916 |
| Streptococcus *equi* subspecies *zooepidemicus* | BCS (1-5) | 0.078 | 0.24 | 1 | 0.105 | 0.746 |
|  | Behaviour signs | 0.229 | 0.094 | 2 | 5.924 | 0.004 |
|  | Nasal discharge presence | 0.138 | 0.105 | 1 | 1.745 | 0.190 |
|  | Coughing presence | 0.216 | 0.088 | 1 | 6.052 | 0.016 |
|  | Sampling moment | 0.453 | 0.224 | 1 | 4.084 | 0.047 |
